# Combined hepatocellular carcinoma–cholangiocarcinoma: A misdiagnosed case preoperatively

**DOI:** 10.1002/jgh3.12574

**Published:** 2021-05-20

**Authors:** Lei Ding, Wang Hu, Qingrong Wu, Zhiqiang Hu, Jianhong Zhang, Lihui Jiang, Zeming Hu

**Affiliations:** ^1^ Department of General Surgery Affiliated Xiaoshan Hospital, Hangzhou Normal University Hangzhou China; ^2^ Department of Pediatric Surgery Ruijin Maternal and Child Health Hospital Ganzhou China; ^3^ Department of Pharmacy Ganzhou Fifth People's Hospital Ganzhou China; ^4^ Department of General Surgery The First Affiliated Hospital of Gannan Medical University Ganzhou China

**Keywords:** combined hepatocellular carcinoma–cholangiocarcinoma, hepatocellular carcinoma, misdiagnosed

## Abstract

Combined hepatocellular carcinoma‐cholangiocarcinoma (cHCC‐CC) is a rare subtype in primary liver cancer, which is difficult to diagnose in clinics. In this report, we present a case of a 62‐year‐old male with abdominal pain and slightly elevated alpha‐fetoprotein. The contrast‐enhanced computed tomography scans showed a left liver mass, which showed adherence to the imaging characteristics of hepatocellular carcinoma. However, with the treatment of laparoscopic liver resection, the following histological examination displayed two distinct components, which were consistent with the diagnosis of cHCC‐CC.

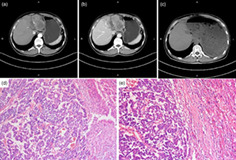

## Case report

**Figure 1:**
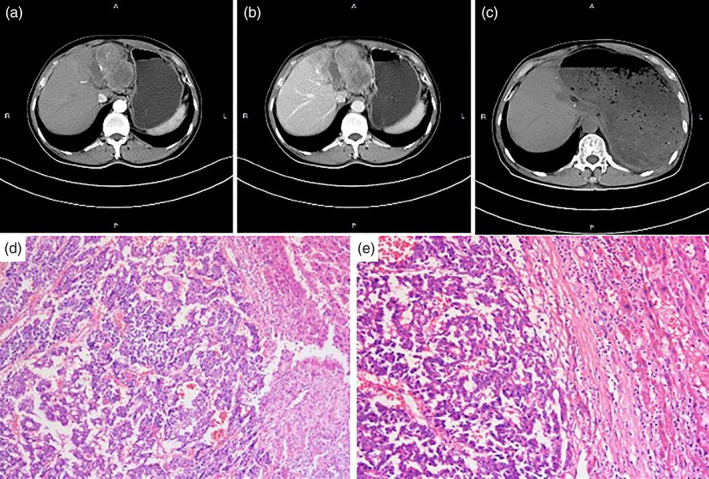
Abdominal contrast‐enhanced computed tomography images displaying a left liver destructive lesion on the hepatic arterial phase (a) and the portal venous phase (b), respectively. (c) Computed tomography of the abdomen revealed postoperative changes with the absence of the left liver lobe. (d and e) HE staining of the specimen (×100, ×200) revealed the tumor displayed two distinct components that were characteristics of hepatocellular carcinoma and cholangiocarcinoma.

A 62‐year‐old man with a 6‐year history of hepatitis B virus infection presented with a 2‐month history of persistent upper abdominal discomfort but worsening for a week, and he was admitted to our department. Laboratory findings revealed his serum alpha‐fetoprotein was mildly elevated at 21, 312 ng/mL, and his hepatitis B serology was positive. Serum liver function tests were within the normal limits except for the slightly elevated alanine aminotransferase (250 U/L) and aspartate aminotransferase (210 U/L). Abdominal contrast‐enhanced computed tomography (CT) scans revealed a destructive lesion that showed enhancement during the hepatic arterial phase (Fig. 1a) and rapid washout during the portal venous phase (Fig. 1b) in the left liver lobe, which was most likely consistent with hepatocellular carcinoma (HCC).

However, the patient has subsequently performed a laparoscopic left hepatectomy and his pathology examination indicated the specimen was poorly differentiated and displayed two distinct components: one with HCC‐like feature and the other with cholangiocarcinoma (CC) pattern (Fig. 1d,e). Further immunohistochemical staining confirmed the diagnosis of combined hepatocellular carcinoma–cholangiocarcinoma (cHCC‐CC). The postoperative course was without severe complications and the patient was discharged 15 days after surgery. During the subsequent follow‐up for 1 year since surgery, the patient remains well without any signs of recurrence (Fig. 1c).

## Discussion

cHCC‐CC is a relatively rare entity of primary liver malignancy, with incidence widely varying among regions.^1^ It is frequently misdiagnosed in the preoperative setting as either HCC or CC because the clinic characteristics and imaging features often mimic those of HCC or CC.^2^ The lesion in the liver shows typical radiological characteristics of HCC, which presents diagnostic challenges of cHCC‐CC to the radiologist and surgeon. Indeed, the recent consensus recommends that immunohistochemistry staining is not a prerequisite for the diagnosis of cHCC‐CC, while that morphology is the key.^3^ Thus, preoperative ultrasound‐ or CT‐guided biopsy and histopathology analysis, especially for the tumor morphology, should be highlighted in diagnosis.
